# Panobinostat reduces hypoxia-induced cisplatin resistance of non-small cell lung carcinoma cells via HIF-1α destabilization

**DOI:** 10.1186/1476-4598-14-4

**Published:** 2015-01-21

**Authors:** Carina Fischer, Katharina Leithner, Christoph Wohlkoenig, Franz Quehenberger, Alexandra Bertsch, Andrea Olschewski, Horst Olschewski, Andelko Hrzenjak

**Affiliations:** Division of Pulmonology, Department of Internal Medicine, Medical University of Graz, Auenbruggerplatz 15, A-8036 Graz, Austria; Red Cross Transfusion Service for Upper Austria, Linz, Austria; Institute of Medical Informatics, Statistics and Documentation, Medical University of Graz, Graz, Austria; Ludwig Boltzmann Institute for Lung Vascular Research, Medical University of Graz, Graz, Austria

**Keywords:** Non-small cell lung cancer, Cisplatin, Panobinostat, Apoptosis, HIF-1α

## Abstract

**Background:**

Lung cancer is one of the most frequent cancer types and the leading cause of cancer death worldwide. Cisplatin is a widely used chemotherapeutic for non-small cell lung carcinoma (NSCLC), however, its positive effects are diminished under hypoxia. We wanted to determine if co-treatment with cisplatin and histone deacetalyse (HDAC) inhibitor panobinostat can reduce hypoxia-induced cisplatin resistance in NSCLC cells, and to elucidate mechanism involved.

**Methods:**

Expression status of different HDACS was determined in two cell lines and in tumor tissue from 20 patients. Cells were treated with cisplatin, panobinostat, or with combination of both under normoxic and hypoxic (1% O_2_) conditions. Cell cycle, viability, acetylation of histones, and activation of apoptosis were determined. HIF-1α stability and its interaction with HDAC4 were analyzed.

**Results:**

Most class I and II HDACs were expressed in NSCLC cells and tumor samples. Co-treatment of tumor cells with cisplatin and panobinostat decreased cell viability and increased apoptosis more efficiently than in primary, non-malignant bronchial epithelial cells. Co-treatment induced apoptosis by causing chromatin fragmentation, activation of caspases-3 and 7 and PARP cleavage. Toxic effects were more pronounced under hypoxic conditions. Co-treatment resulted in destabilization and degradation of HIF-1α and HDAC4, a protein responsible for acetylation and de/stabilization of HIF-1α. Direct interaction between HDAC4 and HIF-1α proteins in H23 cells was detected.

**Conclusions:**

Here we show that hypoxia-induced cisplatin resistance can be overcome by combining cisplatin with panobinostat, a potent HDAC inhibitor. These findings may contribute to the development of a new therapeutic strategy for NSCLC.

**Electronic supplementary material:**

The online version of this article (doi:10.1186/1476-4598-14-4) contains supplementary material, which is available to authorized users.

## Background

Lung cancer is one of the most frequent cancer types in men and women and the leading cause of cancer death worldwide [1]. Prolonged lifespan and exposure to increasing number of new etiologic agents, like tobacco smoke and air pollution, makes lung cancer a menace of the last decades [2]. The most common type is non-small cell lung cancer (NSCLC) with approx. 75% frequency of occurrence, compared to small cell lung cancer (SCLC) with only 25%. Standard therapeutic approaches for NSCLC are surgical resection and adjuvant chemo- and radio-therapy. The majority of patients are not candidates for surgery due to advanced disease at diagnosis. Effectiveness of chemo- and radiotherapy is frequently negatively influenced by microenvironmental factors like hypoxia, which results in a limited increase in survival time. Therefore, alternative and novel molecular-targeted therapeutic approaches are needed. Tyrosine kinase inhibitors targeting the epidermal growth factor receptor (EGFR-TKIs; e.g. erlotinib and gefitinib) markedly improved median survival and quality of life of NSCLC patients with EGFR mutations. However, NSCLCs with K-ras mutations are frequently resistant to EGFR-TKIs [3]. For a subset of NSCLC patients with the echinoderm microtubule protein like-4/anaplastic lymphoma kinase (EML-4/ALK) translocation, crizotinib is approved by the FDA as a second-line therapy. Though, it must be stressed that benefits of those approaches are mostly limited to a specific subgroup of patients [3–6]. Platinum-based therapeutics (e.g. cisplatin and carboplatin) play an important role in the treatment of different human malignancies. Cisplatin (*cis*-diammine-dichloro-platinum) is one of the most potent chemotherapeutic agents against a wide spectrum of solid tumors, including ovarian, bladder, stomach, prostate, and lung carcinoma [7]. However, its positive effects are limited due to acquired drug resistance and severe side effects in non-malignant tissue, especially due to dose-dependent nephro- and/or neuro-toxicity [8, 9]. A growing body of experimental evidence suggests that in lung carcinoma positive cisplatin effects are markedly diminished under hypoxic conditions, a distinctive property of solid tumors, resulting from a misbalanced supply and consumption of oxygen [10–15]. To overcome chemotherapy resistance, platinum-based drugs are combined with antineoplastic drugs targeting other pathways important for tumor growth and progression [16, 17]. Hypoxia-inducible factor HIF-1α is one of the most intensively studied proteins induced by hypoxia. Although its prognostic impact is still not clear, increased expression of HIF-1α correlates with a poorer survival in NSCLC and in patients with other solid tumors [10–18]. Among other mechanisms, HIF-1α stability and activity are regulated by multiple histone deacetylases (HDACs) and can thus be mediated by histone deacetylase inhibitors [19, 20].

Panobinostat is a cinnamic hydroxamic acid analogue that affects both class I and class II HDACs. Acetylation upon panobinostat treatment also occurs on non-histone cytoplasmic proteins, e.g. α-tubulin, HIF-1α, HSP90, and p53 [21]. Panobinostat induces a growth arrest, terminal differentiation and/or apoptosis. It has also been shown, both *in vitro* and *in vivo*, to have antitumorigenic effects in a variety of cancer cell lines (NSCLC, prostate, colon, ovary, breast, lymphomas, etc.) [22–24].

In the present study we investigated the impact of co-treatment with cisplatin and panobinostat on survival of NSCLC cell lines under normoxic and particularly under hypoxic conditions. To our knowledge, this is the first study showing that hypoxia-induced cisplatin resistance in NSCLC cells can be overcome by co-treatment with panobinostat via destabilization of HIF-1α.

## Results

### Class I and II HDACs are expressed in NSCLC cell lines and lung cancer tissue

**Figure 1:**
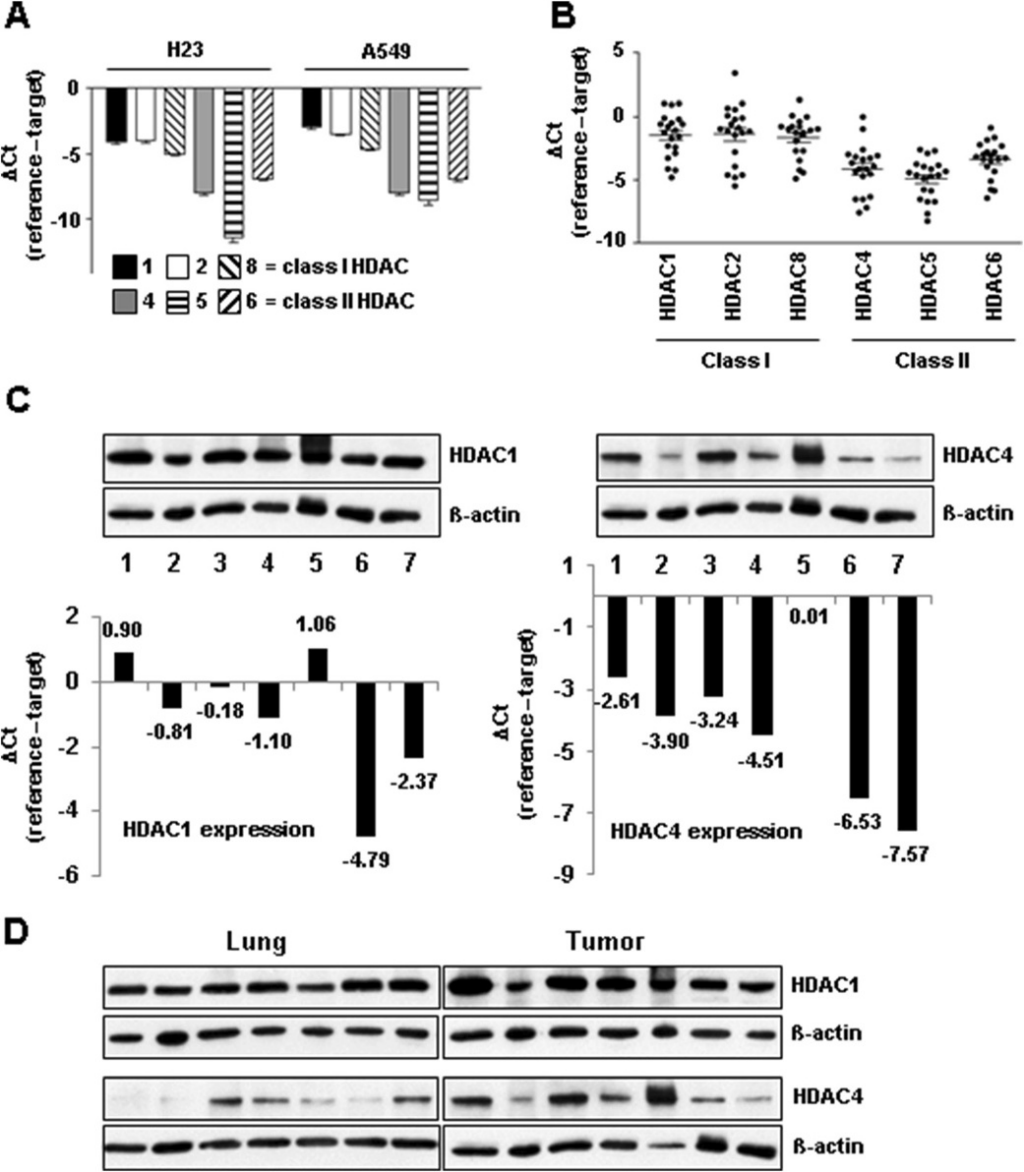
**Expression levels of class I and II HDACs in NSCLC cell lines and tumor tissue. (A)** RNA was isolated from H23 and A549 cells, transcribed in cDNA and expression levels were analyzed by qRT-PCR. For normalization β-actin was used as a reference and data were presented as mean ΔCt values ± SEM. **(B)** RNA was isolated from fresh post-operative lung carcinomas. After reverse transcription, qRT-PCR was performed and expression levels of different HDACs in tumor tissue were normalized to β-actin and data were presented as mean ΔCt values ± SEM. **(C, D)** For protein analysis cryo-preserved lung tumor and non-malignant lung tissue was homogenized by blender, followed by sonication (3 x 5 seconds on ice) and centrifugation at 13000 rpm for 10 min. at 4°C in order to remove insoluble cell-debris. Tissue lysates (20 μg total protein) were analyzed by immunoblotting for expression of HDAC1 and HDAC4 in different tumor samples **(C)** and in comparison to corresponding non-malignant lung tissue **(D)**. **(C)** Corresponding mRNA expression data are shown. ß-actin = loading control.

The expression levels for three members of class I HDAC (HDAC 1, 2 and 8) and three members of class II HDAC (HDAC 4, 5 and 6) were analyzed by quantitative real-time polymerase chain reaction (qRT-PCR) in two NSCLC cell lines, as well as in lung cancer tissue from twenty patients. In both cell lines (H23 and A549) all six HDACs were expressed, HDACs 1 and 2 showing the highest, and HDAC5 the lowest expression level (Figure 1A). Twenty lung cancer tissue samples showed a similar expression pattern (Figure 1B). Compared to class I, the expression levels of class II HDACs were slightly lower, both in cell lines and in tumor tissue. Cryo-preserved tissue samples used in this study represent three histological NSCLC subtypes: adenocarcinoma (n = 9), squamous cell carcinoma (n = 7), and large cell carcinoma (n = 4). The level of expression of different HDACs was comparable between different subtypes, with slight tendency of elevated expression levels in squamous cell carcinoma (Additional file 1).Immunoblotting with total cell lysates from frozen cancer tissue samples (n = 7) showed correlation between the RNA levels and the protein expression levels for representative members (HDAC1 and HDAC4) of class I and class II HDACs (Figure 1C). As expected, there was a certain biological diversity in different patients. The observed variability of HDAC4 expression was larger than of HDAC1. We also analyzed the expression of HDAC1 and HDAC4 proteins in lung tumors and corresponding non-malignant lung tissue from seven patients (Figure 1D). These data suggest that the protein level of both HDAC1 and HDAC4 is slightly higher in lung tumors than in non-malignant lungs. However, for more accurate conclusions a higher number of matched samples is obligatory.

### Panobinostat inhibits proliferation of NSCLC cells

**Figure 2:**
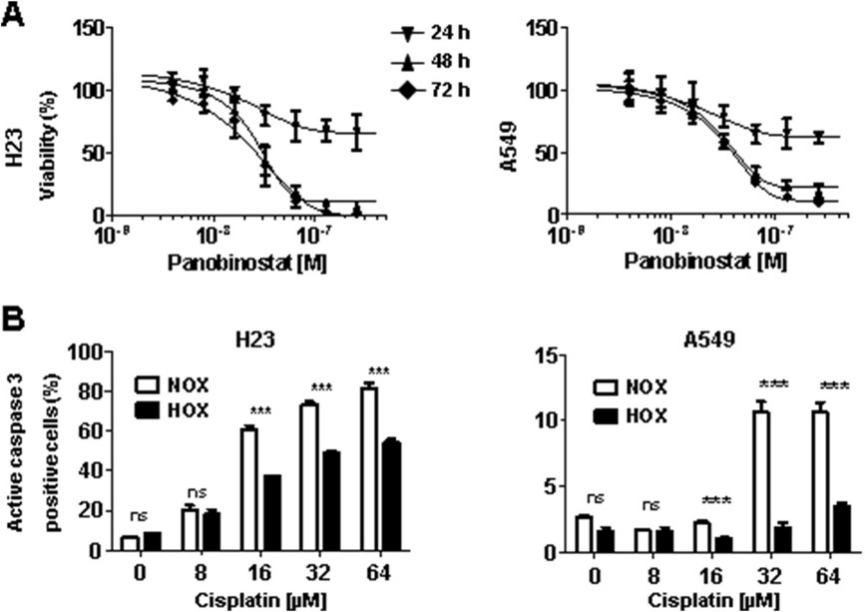
**Cell viability upon treatments under normoxic (NOX) and hypoxic (HOX) conditions. (A)** H23 and A549 cells were pre-incubated for 24 hours under normoxic conditions (21% O_2_), treated for indicated time intervals with panobinostat (4, 8, 16, 32, 64, 128 and 256 nM; 1 mM stock solution in DMSO) and finally the cell viability was measured using the AlamarBlue® assay. DMSO was used as a vehicle control. All results were compared to DMSO-treated cells set to 100%. **(B)** H23 and A549 cells pre-incubated under specific conditions for 24 hours were treated with increasing cisplatin concentrations (dissolved in 0.9% NaCl) and apoptosis was measured by the PhiPhi-Lux® assay detecting active caspase-3. NOX, normoxia; HOX, hypoxia; ns, not significant; *** *P* < 0.001.

Treatment with panobinostat induced dose- and time-dependent inhibition of cell viability in both, H23 and A549 cell lines (Figure 2A) with calculated IC_50_ values (for 48 and 72 hours treatment) in a low nanomolar range (14 nM to 30 nM), which is in line with published data [25]. H23 cells showed higher sensitivity to panobinostat than A549 cells, especially after 48 and 72 hours of treatment. We used panobinostat concentrations which are in line with published studies, where concentrations were ranging from 12–100 nM, the average concentration being approx. 50 nM [26, 27]. H23 and A549 cells were treated under normoxic (NOX, 21% O_2_) and hypoxic (HOX, 1% O_2_) conditions with cisplatin concentrations which have been used in previous studies and were described as clinically relevant [28, 29]. The results for apoptosis activation showed very pronounced, highly significant hypoxia-induced cisplatin resistance (Figure 2B). This is in line with published data showing that under hypoxic conditions NSCLC cells develop cisplatin resistance [15, 30].

### Co-treatment with cisplatin and panobinostat has superior inhibitory effects on tumor cell viability

**Figure 3:**
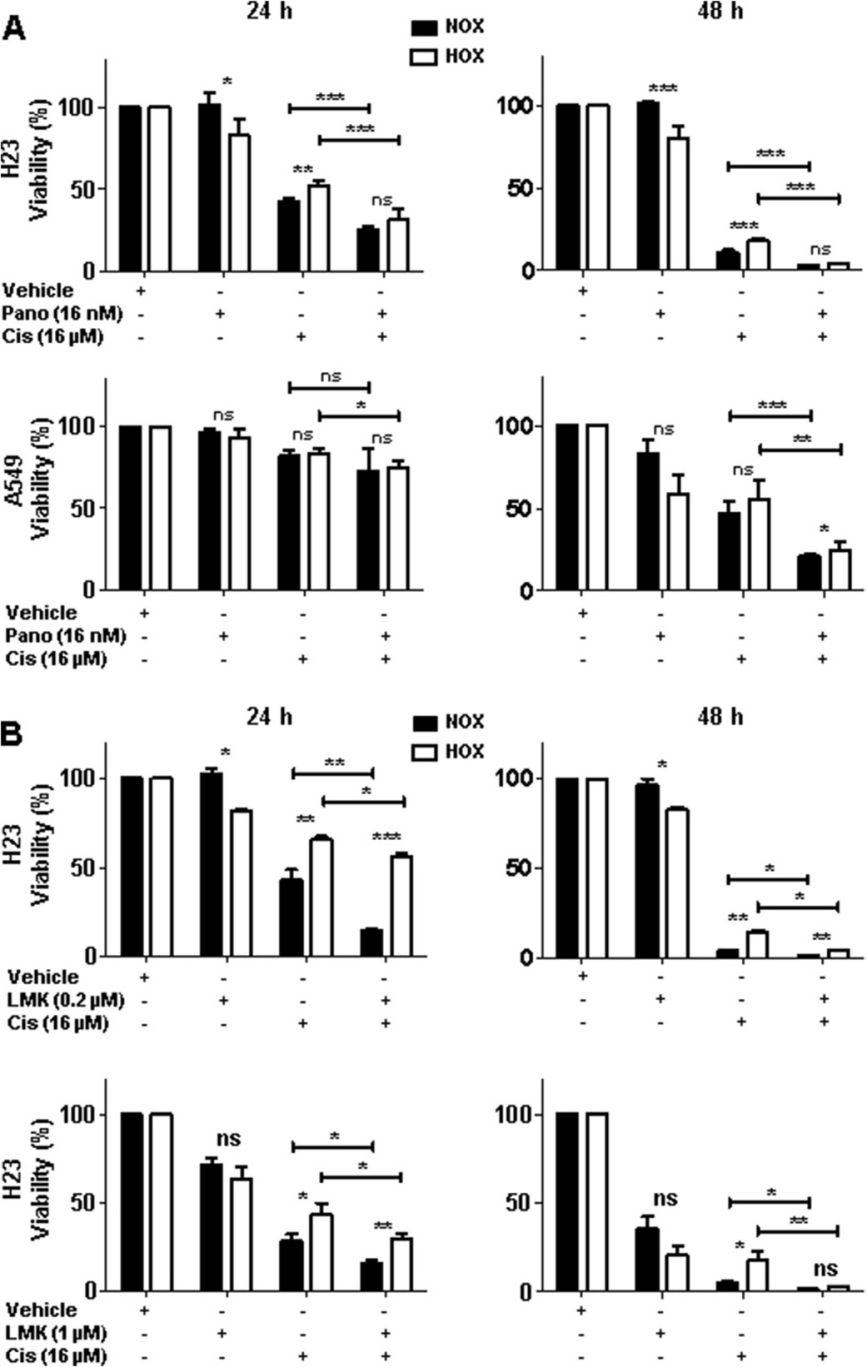
**Effects of co-treatment with cisplatin and panobinostat on cell viability. (A)** Cells were treated with 16 μM cisplatin or 16 nM panobinostat alone and with combination of both substances for 24 and 48 hours. Afterwards cell viability was determined with the AlamarBlue® assay (n ≥ 6 measurements per each condition). DMSO was used as a vehicle control and all results were compared to DMSO-treated cells set to 100%. **(B)** H23 cells were treated with 0.2 μM and 1 μM LMK235 (N-((6-(hydroxyamino)-6-oxohexyl)oxy)-3,5-dimethylbenzamide) alone and in combination with cisplatin for 24 and 48 hours. LMK235 is a new HDAC inhibitor selective towards human HDAC4 and 5. Cell viability was determined with AlamarBlue® assay and results from three independent experiments (n = 4 measurements per each condition) were compared to DMSO-treated cells set to 100%. NOX, normoxia; HOX, hypoxia; ns, not significant; * *P* < 0.05; ** *P* < 0.01; *** *P* < 0.001; determined by Student’s t-test. Pano, panobinostat; Cis, cisplatin.

We investigated whether treatment with cisplatin in combination with panobinostat may overcome hypoxia-induced cisplatin resistance of NSCLC cells. H23 and A549 cells were treated for 24 and 48 hours with single substances or with combination of both under normoxic and hypoxic conditions. To investigate effects of both substances, cells were simultaneously treated with panobinostat (16 nM) and cisplatin (16 μM) and those results were statistically compared to results for cisplatin treatment alone. Compared to the cisplatin-induced growth inhibition, in H23 cells there was a significant decrease of cell viability caused by co-treatment at 24 (*P* < 0.001) and 48 hours (*P* < 0.001) (Figure 3A). Importantly, these effects were also very pronounced under hypoxic conditions, where cisplatin alone caused significantly lower cytotoxic effects. The combination with a lower cisplatin concentration (8 μM) and higher panobinostat concentration (32 nM) also revealed higher cell death in comparison to single cisplatin treatment (Additional file 2). After 24 hours treatment the cell viability of A549 cells was not decreased to the same extent as for H23 cells. However, after 48 hours treatment there was a significant difference in favor of co-treatment, both under normoxic (*P* < 0.001) and hypoxic (*P* < 0.01) conditions. In A549 cells these effects were significant for both co-treatments (16 nM panobinostat and 16 μM cisplatin; 32 nM panobinostat and 8 μM cisplatin) (Figure 3A, Additional file 2). Most importantly, in both cell lines and under both conditions the cell viability upon co-treatment was significantly lower compared to treatment with cisplatin alone.

In H23 cells a synergistic effect (negative sign of the mean difference from additivity) was observed for both dose combinations (16 nM panobinostat and 16 μM cisplatin, 32 nM panobinostat and 8 μM cisplatin) under both oxygenation conditions (Additional file 3). After 24 hours under normoxic conditions statistically significant synergy was observed for the combination 16 nM panobinostat and 16 μM cisplatin. After 48 hours statistical significance was obtained in all assays. To further investigate inhibitory effects, we pre-treated cells with panobinostat for 24 hours, followed by cisplatin treatment for another 24 hours. In terms of cell viability this consecutive treatment did not show any benefits in comparison to simultaneous co-treatment (data not shown).

We treated H23 cells with another class II HDACs inhibitor, LMK235 (N-((6-(hydroxyamino)-6-oxohexyl)oxy)-3,5-dimethylbenzamide), which has a unique selectivity towards human HDAC4 and 5 [31]. LMK235, both alone (0.2 and 1 μM) and in combination with 16 μM cisplatin, caused similar effects as panobinostat (Figure 3B).

### Co-treatment inhibits growth of multicellular spheroids (MCS)

**Figure 4:**
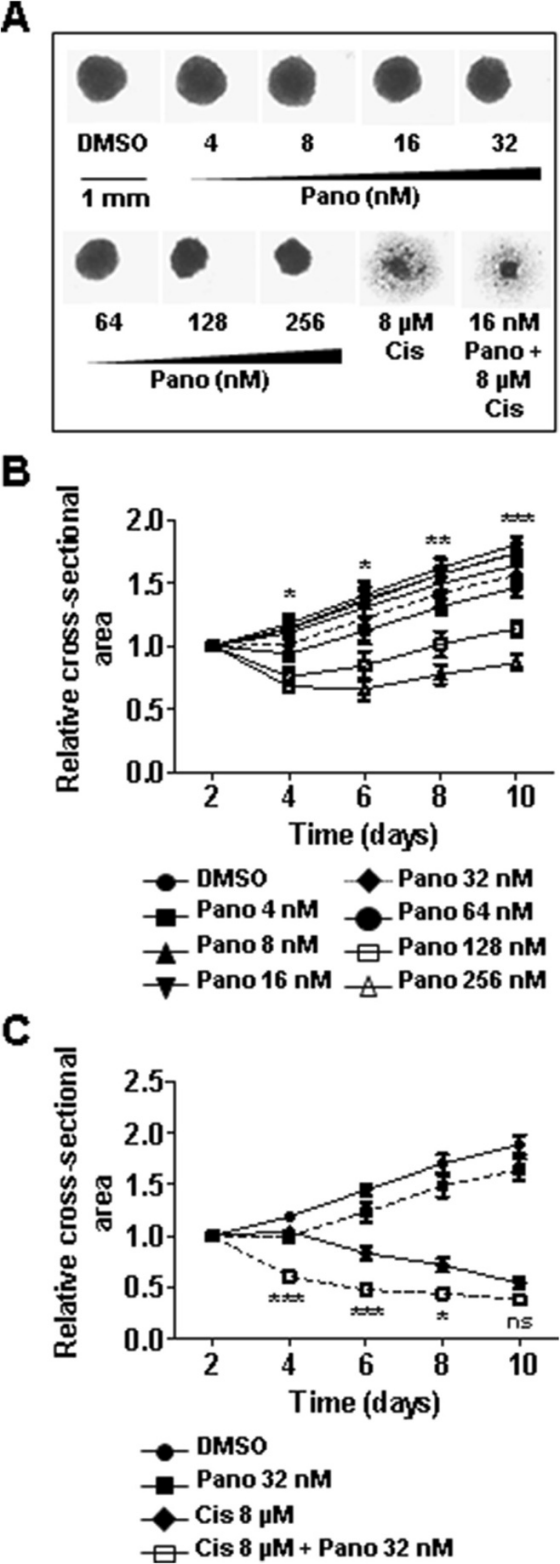
**Effects of co-treatment on growth of multicellular spheroids (MCS). (A)** Multicellular spheroids were prepared as described in Materials and methods. After treatment with indicated concentrations of panobinostat, cisplatin or with combination of both for 24 hours, medium was replaced and spheroids were cultivated under standard normoxic conditions. MCS size was measured every second day for 10 days and on day 10 photographs were made. **(B)** MCS were incubated with indicated panobinostat concentrations. MCS size was measured every second day over 10 days and the relative cross-sectional area (single time-point values normalized to MCS size on day of treatment = day 2) were determined. Group comparisons were performed with Two-way ANOVA and Bonferroni post-hoc analysis. The significances for panobinostat (32 nM) vs. DMSO are shown. **(C)** After treatment with single drugs or with combination of both the MCS size was determined as already described. The significances for cisplatin vs. cisplatin + panobinostat are shown. Pano, panobinostat; Cis, cisplatin; ns, not significant; * *P* < 0.05; ** *P* < 0.01; *** *P* < 0.001.

To better mimic the hypoxic microenvironment present in lung carcinoma, we used the MCS model where hypoxic conditions develop spontaneously because of limited oxygen diffusion. MCS were prepared as described in Materials and methods, incubated for two days in order to adapt to *in vitro* conditions and treated with indicated concentrations of panobinostat, cisplatin or a combination of both. Size measurements performed on every second day showed a concentration-dependent reduction of MCS size upon panobinostat treatment (Figure 4A). Two days upon treatment (day 4) size reduction of 43% between vehicle control and MCS treated with 256 nM panobinostat was observed. In consecutive measurements this reduction settled down to approx. 53% (Figure 4B). Co-treatment with 16 nM panobinostat and 8 μM cisplatin induced reduction of MCS size to 57% on day 2 and remained at a similar level with slightly milder effects on day 10 (70%) (Figure 4C). These data indicate that panobinostat enhanced the effect of cisplatin treatment.

### Co-treatment triggers chromatin fragmentation and induction of apoptosis

**Figure 5:**
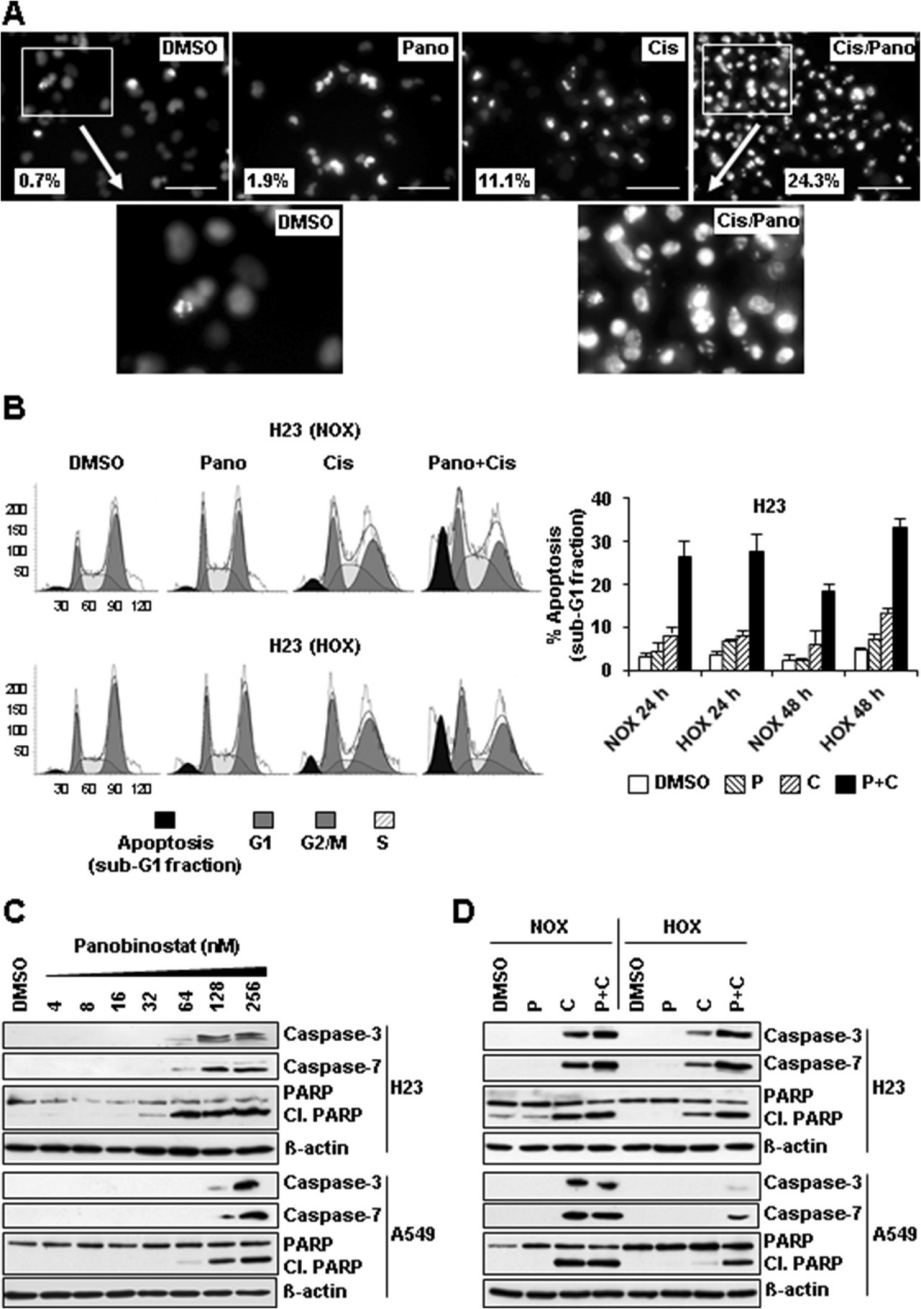
**Activation of apoptosis in NSCLC cells upon co-treatment with cisplatin and panobinostat. (A)** H23 cells treated for 24 hours with single substances or with combination of both were stained with Hoechst 33342 in order to determine the level of chromatin fragmentation as an indicator of apoptosis. Cells were observed under the fluorescent microscope and representative photographs were made. Note markedly increased chromatin fragmentation in co-treated cells, those effects being better visible in boxed area at higher magnification. Percentage of fragmented nuclei in relation to total number of cells is shown (three independent experiments performed in triplicates). Scale bar = 50 μm. **(B)** Upon treatment H23 cells were stained with propidium iodide (PI) and analyzed by FACS for the sub-G1 peak characteristic for apoptosis. Data analysis was performed by ModFit LT 3.3 software package. Representative figures (left panel) and mean values (right panel) are shown. **(C, D)** NSCLC cells were treated with indicated concentrations of panobinostat, cisplatin or with combination of both, harvested in RIPA buffer and cell lysates were analyzed by immunoblotting using antibodies against different apoptosis markers. Upon stripping β-actin was used as loading control. P, panobinostat (16 nM); C, cisplatin (16 μM); P + C (16 nM panobinostat + 16 μM cisplatin); NOX, normoxia; HOX, hypoxia; Cl. PARP = cleaved PARP.

Apoptosis-induced chromatin fragmentation in H23 and A549 cells was analyzed by Hoechst 33342 staining (Figure 5A). At low concentrations (16 nM) panobinostat only slightly induced chromatin fragmentation in both cell lines. As expected, cisplatin (16 μM) triggered fragmentation of chromatin. However, those effects were significantly (P < 0.01) stronger upon co-treatment (16 μM cisplatin and 16 nM panobinostat). Chromatin fragmentation was very pronounced in H23 cells and slightly weaker in A549 (data not shown).

We further analyzed the sub-G1 peak, as an indicator of apoptosis. After different treatments, cells were stained with propidium iodide and cell cycle analysis was performed for H23 cells (Figure 5B) and for A549 cells (Additional file 4). Upon 24 hours treatment with a low panobinostat concentration (16 nM) only slightly increased apoptosis was observed, whereas cisplatin-induced apoptosis was more pronounced. In line with chromatin fragmentation data generated by Hoechst 33342 staining, the apoptotic rate in co-treated cells was markedly increased, being in a range between 18-32% of all gated cells. In H23 cells apoptotic rate under hypoxic conditions was equal or even higher than under normoxic conditions. In A549 cells similar, although milder effects were observed. By cell cycle analyses we detected a weak G1 arrest in H23 cells treated with cisplatin and with cisplatin plus panobinostat (Additional file 5).In panobinostat treated cells, cleavage (activation) of caspases-3 and 7 as well as cleavage of PARP (poly(ADP-ribose) polymerase, 89 kDa) was detected primarily in cells treated with higher concentrations; 64 to 256 nM in H23 and 128 to 256 nM in A549, respectively (Figure 5C). These results confirm our cell viability data showing that A549 cells are slightly less sensitive to panobinostat than H23 cells. Low panobinostat concentration (16 nM) did not induce apoptosis, those effects being similar under normoxic and hypoxic conditions (Figure 5D). All three apoptotic markers were elevated by cisplatin treatment under both conditions. However, effects under hypoxic conditions were less pronounced, indicating hypoxia-induced cisplatin resistance. In co-treated cells cleavage of all three markers was increased to higher extent than by cisplatin alone, suggesting higher efficacy of this combination. Most importantly, those effects were more pronounced under hypoxic conditions. Altogether, these data suggest that cell type-specific time- and concentration-dependent growth inhibition, especially in co-treated cells, primarily results from increased induction of apoptosis.

### Non-malignant bronchial epithelial cells are more resistant to co-treatment than NSCLC tumor cells

**Figure 6:**
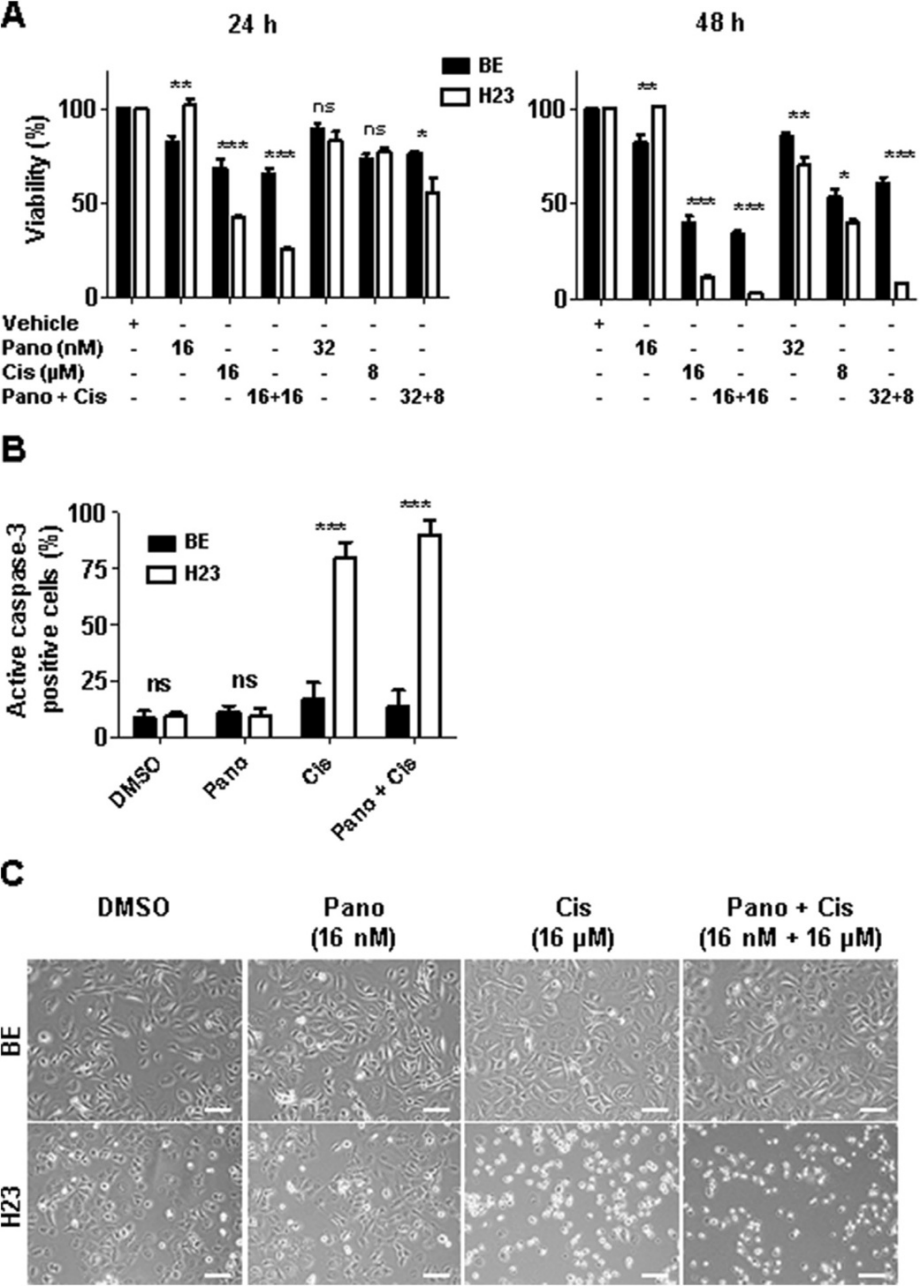
**Effects of co-treatment on non-malignant bronchial epithelial cells. (A)** Bronchial epithelial cells (BE) were isolated from explanted healthy human lungs (see Materials and methods for details) and kept in cell culture under normoxic conditions up to 4th passage. H23 and BE cells were treated with panobinostat, cisplatin or combination of both for 24 or 48 hours, followed by cell viability measurement. **(B)** Upon different treatments of H23 and BE cells for 48 hours the Caspase-3 Intracellular Activity Assay Kit I (PhiPhi-Lux®) was performed, followed by FACS analysis. Data are presented as percentage of cells positive for active caspase-3. **(C)** Cell morphology of non-malignant BE and malignant H23 cells 48 hours upon different treatments. Scale bar = 100 μm. ns, not significant; * *P* < 0.05; ** *P* < 0.01; *** *P* < 0.001; determined by Student’s t-test. Pano, panobinostat; Cis, cisplatin.

Primary, non-malignant bronchial epithelial (BE) cells were isolated from explanted human lungs in our laboratory and cultured under normoxic conditions. Co-treatment (16 nM panobinostat and 16 μM cisplatin) over 24 hours showed a significantly higher survival of BE cells (66%) in comparison to malignant H23 cells (25%) (P < 0.001) (Figure 6A). These effects were even more pronounced after 48 hours co-treatment of BE (34%) and H23 (3%) cells, respectively. The combination of a lower cisplatin concentration (8 μM) and a higher panobinostat concentration (32 nM) showed milder effects after 24 hours, but after 48 hours strong inhibition of cell viability in favor of H23 cells was observed (8% viability for H23 versus 60% for BE cells). After the co-treatment the number of BE cells positive for caspase-3, as an indication of apoptosis, was markedly lower in comparison to H23 cells (13.8% vs. 90% in BE and H23 cells, respectively; P < 0.001) (Figure 6B). These effects were also reflected in the cell morphology (Figure 6C). Altogether, malignant NSCLC cells showed markedly increased sensitivity to the co-treatment in comparison to non-malignant BE cells.

### Co-treatment increases histone-acetylation and promotes strong destabilization of HIF-1α correlated with HDAC4 down-regulation

**Figure 7:**
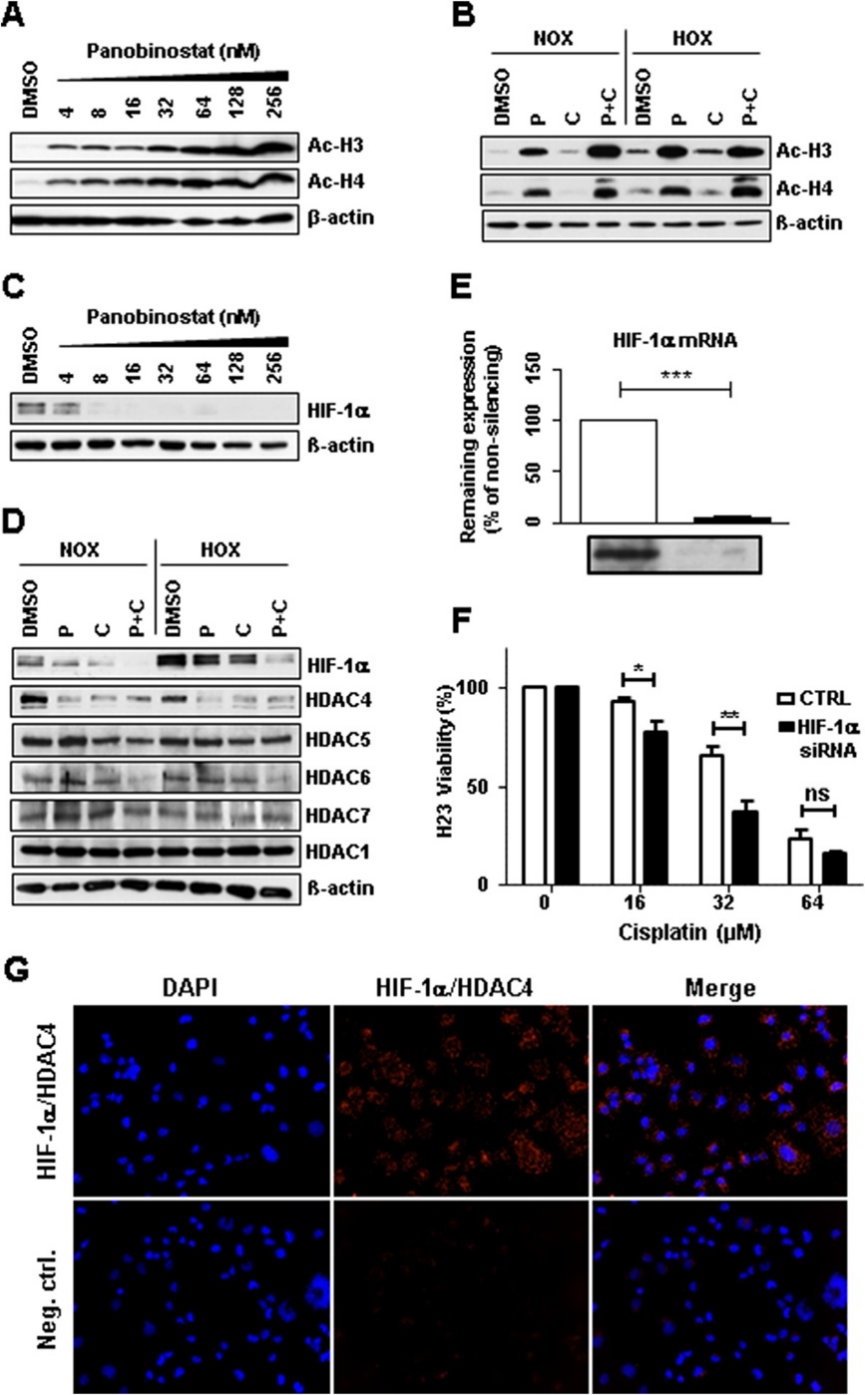
**Identification of histone-acetylation and HIF-destabilization in H23 cells upon co-treatment with panobinostat and cisplatin. (A)** H23 cells were treated with increasing panobinostat concentrations under normoxic conditions and the amount of acetylated histone proteins H3 and H4 was detected by immunoblotting. **(B)** H23 cells were treated with different combinations of cisplatin or/and panobinostat both under normoxic (NOX) or hypoxic (HOX) conditions. The amounts of Ac-H3 and Ac-H4 were analyzed 24 hours later by immunoblotting. **(C)** After 24 hours treatment with increasing panobinostat concentrations immunoblotting for HIF-1α and HIF-2α was performed. **(D)** H23 cells were treated with cisplatin, panobinostat or with combination of both under normoxic and hypoxic conditions. After 24 hours immunoblotting for HIF-1α, class II HDACs (HDAC4, 5, 6 and 7) and HDAC1 (class I) was performed. After stripping β-actin was used as a loading control. Ac-H3 and H4, acetylated histone proteins; DMSO, vehicle control; P, panobinostat (16 nM); C, cisplatin (16 μM); P + C (16 nM panobinostat + 16 μM cisplatin); NOX, normoxia; HOX, hypoxia. **(E)** H23 cells were transfected with 10 nM of HIF-1α siRNA and after 24 hours knock-down effects were detected by qRT-PCR and by immunoblotting. **(F)** H23 cells transfected with HIF-1α siRNA were treated with different cisplatin concentrations for 24 hours under hypoxic conditions and cell viability was measured using the AlamarBlue® assay. Viability of untreated cells was set to 100%. Data were compared to cells transfected with control, non-silencing RNA (CTRL). ns, not significant; * *P* < 0.05; ** *P* < 0.01; *** *P* < 0.001. **(G)** Protein-protein interaction between HIF-1α and HDAC4 was determined by Duolink *in situ* proximity ligation assay and visualized by fluorescence microscopy. For negative control primary antibodies were omitted. Magnification: 200x.

Acetylation of histones 3 and 4 (Ac-H3, Ac-H4) was analyzed in order to predict chromatin condensation level. Panobinostat induced concentration-dependent accumulation of Ac-H3 and Ac-H4 (Figure 7A, Additional file 6A). In co-treated cells this accumulation was further increased, especially for Ac-H4, both under normoxic and under hypoxic conditions (Figure 7B). To characterize the molecular mechanism underlying cell viability decrease and activation of apoptosis upon co-treatment, we analyzed protein level of the hypoxia-induced factor 1α (HIF-1α), a major regulatory protein involved in cell adaptation to hypoxia and development of hypoxia-induced cisplatin resistance. Panobinostat markedly decreased the amount of HIF-1α in H23 cells in a concentration-dependent manner (Figure 7C, Additional file 6B). As expected, the amount of HIF-1α under hypoxic conditions was generally much higher, indicating characteristic hypoxia-mediated HIF-1α stabilization. Most importantly, in both cell lines the amount of HIF-1α in co-treated cells was almost abolished, both under normoxic and especially under hypoxic conditions (Figure 7D, Additional file 6C and D). We also analyzed relative expression level of HIF-1α by qRT-PCR, but on mRNA level there was no difference between cells treated with single drugs or with the combination of both (data not shown). Since in the literature there is a link between de/stabilization of HIF-1α protein and expression of HDAC4, we analyzed expression of HDAC4 upon different treatments. In co-treated H23 cells the expression of HDAC4 was decreased in a similar way as HIF-1α (Figure 7D). Expression levels of other class II HDAC members (HDAC5, 6 and 7) were not influenced in the same way as HDAC4 and did not correlate with HIF-1α (Figure 7D). HDAC9 was not detectable by immunoblotting, which corresponds to a recent study showing significant down-regulation of HDAC9 in lung cancer cells and lung tumor tissue [32]. The expression level of HDAC1, a class I HDAC member known to be highly expressed in lung tissue, was not influenced by single treatments or by the co-treatment with panobinostat and cisplatin.

To further analyze functional consequences of HIF-1α destabilization, we down-regulated HIF-1α expression (up to 95% knock-down) by using a pool of siRNAs containing four HIF-1α-specific siRNA sequences (Figure 7E). Upon transfection and consecutive cisplatin treatment under hypoxic conditions, cell viability was measured and compared to control cells transfected with non-silencing RNA. Our results showed decreased cell-viability in H23 cells transfected with HIF-1α siRNA, clearly demonstrating the central role of HIF-1α in hypoxia-induced cisplatin resistance (Figure 7F). Data generated by *in vitro* proximity ligation assay indicate protein-protein interactions between HDAC4 and HIF-1α (Figure 7G). Interestingly, down-regulation of HDAC4 expression (up to 70% knock-down) by specific siRNA pool did not affect the cisplatin-related cell toxicity (data not shown), indicating a possible interplay and/or redundancy of other HDAC members.

## Discussion

Hypoxia-induced cisplatin resistance is one of the major problems in the therapy of various solid tumors, especially of ovarian and NSCLC cancer [13, 33–35]. Here we hypothesized that, compared to cisplatin alone, co-treatment with the histone deacetylase inhibitor panobinostat induces higher pro-apoptotic and anti-proliferative activity in NSCLC cells. The pan-HDAC inhibitor panobinostat has been evaluated so far in early clinical studies in patients with a variety of hematologic and solid tumors e.g. Hodgkin lymphoma, multiple myeloma, pancreatic cancer, and NSCLC [36, 37]. In different cancer cell lines, co-treatments with panobinostat induced significantly better antitumor effects than single-drug treatments, leading to cumulative or synergistic effects [25, 34, 37–39]. It has been reported that co-treatment with cisplatin and panobinostat reduced cisplatin resistance of ovarian cancer cells [23]. However, no data exist about the co-treatment with cisplatin and panobinostat in NSCLC cells under hypoxic conditions. Our data indicate that under normoxic and hypoxic conditions, different NSCLC cell lines have different sensitivities to panobinostat. Crisanti *et al.* have shown different response rates to panobinostat in eleven NSCLC cell lines under normoxic conditions, with IC_50_ values between 5 and 310 nM, which is consistent with our data for H23 and A549 cells [25]. It must be stressed that commercially available NSCLC cell lines are very heterogeneous regarding genetic defects [40]. Histone deacetylation plays a fundamental role in the proliferation of tumor cells and frequently leads to induction and activation of tumor suppressive genes, including p53 [41]. Deregulated expression of p53 plays a significant role in the development of cisplatin resistance, since several genes implicated in drug resistance and apoptosis are known to be regulated by p53 [42]. While A549 cells express wild-type p53, H23 cells express the mutant p53 protein with a missense mutation in codon 246 (ATC into ATG; isoleucine into methionine) of the p53 gene. Thus, the p53 mutation status might explain, at least partially, differences in panobinostat sensitivity between A549 and H23 cells. That does not diminish the impact of the pan-HDAC inhibitor panobinostat, because it has been suggested that the efficacy of HDACIs with narrower specificity more likely depends on functional p53 [43].

Our findings indicate that, although panobinostat alone shows similar efficacy under normoxic and hypoxic conditions, co-treatment with panobinostat and cisplatin reduced NSCLC cell viability significantly better than cisplatin treatment alone, especially under hypoxic conditions. HDAC inhibitors not only kill proliferating tumor cells but also non-malignant cells, those effects being concentration dependent [44, 45]. Our results clearly demonstrate that primary, non-malignant bronchial epithelial cells are significantly less sensitive to co-treatment than malignant NSCLC cells. This might be of special importance for clinical studies and applications, where side effects on non-malignant cells must be avoided.

So far contradictory data have been published regarding panobinostat-induced G2/M arrest. Prystowsky *et al.* reported that panobinostat causes G2/M cell cycle arrest in head and neck squamous cell carcinomas (HNSCCs) [46]. On the other hand, Ocio *et al.* showed that in multiple myeloma cells the G2/M arrest was not induced by single panobinostat treatment at a low nanomolar concentration (7 nM), [37] while Crisanti *et al.* showed in small-cell lung carcinoma (SCLC) cells that panobinostat increases the sub-G1 peak over a period of 96 hours [25]. In our study, there was no significant G2/M arrest in H23 and A549 cells treated with 16 nM panobinostat. These data indicate that panobinostat-caused G2/M cell cycle arrest is a cell type-specific phenomenon, which does not necessarily determine the response rate to panobinostat in different cell lines. In our cell models, the cell response was rather determined by activation of apoptosis then by influencing the cell cycle.

It is highly interesting that, in our experimental setup, as low as 16 nM panobinostat in combination with cisplatin induced apoptosis significantly better than cisplatin alone. These data indicate pronounced activation of apoptosis in NSCLC cells co-treated with clinically relevant concentrations of cisplatin and panobinostat. We also demonstrate that, especially under hypoxic conditions, treatment of NSCLC cells with panobinostat causes strong acetylation of core histones (H3 and H4). It is well known that these processes modify chromatin compaction, thereby sensitizing cells to treatment with DNA-damaging agents [47].

HIF-1α is one of the best described regulatory gene and transcription factor activated under hypoxia and plays an important role in activation of resistance mechanisms to anticancer drugs [48]. Bohonowych *et al.* have reported that upon treatment with panobinostat the expression level of HIF-1α in renal carcinoma cells was decreased [49]. Here we showed that panobinostat markedly decreased the amount of HIF-1α protein in hypoxic NSCLC cells in a concentration-dependent manner. Most importantly, these effects were most pronounced in hypoxic co-treated cells, where HIF-1α expression was highly abolished by the co-treatment. This is in agreement with decreased cell viability and increased activation of apoptosis. Among other mechanisms, stabilization of HIF-1α is mediated by activity of class II HDACs, especially by HDAC4 [19, 20]. It has been reported that HDAC4 is upregulated in cisplatin-resistant ovarian cancer cells [50]. De Cecco *et al.* showed in a recent study that enforced expression of hsa-miR-302b, targeting HDAC4 gene in ovarian carcinoma cells, significantly enhanced cisplatin cytotoxicity [51]. Inhibition of HDAC4 in kidney cancer cells by specific short hairpin RNA resulted in increased acetylation and degradation, as well as reduced transcriptional activity of HIF-1α, as a result of HDAC4 reduction and subsequent acetylation of N-terminal lysines [19]. Our results, based on the *in vitro* proximity ligation assay, clearly indicate protein-protein interaction between HDAC4 and HIF-1α in H23 cells. We show that, especially under hypoxic conditions, HIF-1α destabilization and degradation upon co-treatment correlates with down-regulation of HDAC4, but not with the expression pattern of other class II HDACs (e.g. HDAC5, 6 and 7). Results with LMK235, an HDAC inhibitor selective towards human HDAC4 and 5, also support our observation that inhibitory effects are rather related to HDAC4 and not to HDAC5, which expression in H23 cells was not influenced by panobinostat. Interestingly, in H23 cells down-regulation of HDAC4 by specific siRNA molecules did not result in destabilization of HIF-1α or decreased cell viability. This could mean that: i) activity of HDAC4 may be more important for HIF stabilization than the HDAC4 expression level; ii) other HDACs can substitute the role of HDAC4 in stabilizing the HIF-1α in NSCLC cells. It should be stressed that panobinostat is a pan-HDAC inhibitor and further studies are necessary, especially in lung-cancer cells, to investigate other HDACs involved in the regulation and stabilization of HIF proteins.

Based on our results, we suggest two possible explanations for strong additive effects of co-treatment with panobinostat and cisplatin in NSCLC cells. First; panobinostat induces hyperacetylation of core histones (H3 and H4) leading to subsequent relaxation of nuclear chromatin. Thus, the logical consequence is a higher accessibility of chromatin for drugs interacting with DNA, such as cisplatin. This is in line with data published for some other cell lines [47]. Second; panobinostat-mediated down-regulation of different HDACs (e.g. HDAC4) and consequent destabilization of HIF-1α under hypoxic conditions increases toxic effects of the co-treatment. In any case, there are still gaps that need to be filled in order to fully understand how HDAC inhibitors can influence tumor-cell growth, either as a single substance or in combination with other drugs.

## Conclusions

Overall, in this study, we clearly showed that in NSCLC cells, cytotoxic effects of cisplatin can be enhanced by co-treatment with the HDAC inhibitor panobinostat. Although these effects are also present under normoxic conditions, they are of special importance under hypoxic conditions, where hypoxia-induced cisplatin resistance is a severe problem. To achieve those positive effects, panobinostat concentrations in a lower nanomolar range are needed; thus, very mild side effects are to be expected**.** These data may initiate development of new therapeutic strategies for chemotherapy of NSCLC patients.

## Materials and methods

### Cultivation of NSCLC cell lines under normoxic and hypoxic conditions

Human NSCLC cell line NCI-H23 (H23) was purchased from American Type Culture Collection (ATCC®, Manassas, VA). Human NSCLC cell line A549 was purchased from Cell Lines Service (Eppelheim, Germany). A549 cells were cultured in DMEM/F-12 with 2 mM L-glutamine and H23 in RPMI 1640 medium, both supplemented with 10% heat-inactivated FCS, 100 U/ml penicillin, and 100 μg/ml streptomycin. Under normoxic conditions (21% O_2_) all cell lines were cultured at 37°C under 5% CO_2_ and 98% humidity. Experiments under hypoxic conditions were performed at 37°C under 5% CO_2_ and 1% O_2_ in the automated Xvivo system G300CL (BioSpherix, Lacona, NY). A gas mixture of N_2_ and CO_2_ (Air Liquide, Paris, France) was connected to the system to hold on a constant O_2_ of 1%, both in the working chamber and in the incubators. Before specific treatment cells were pre-incubated for 24 hours under normoxia or hypoxia. Cell numbers were determined by CASY® (Innovartis, Reutlingen, Germany). For cell authentication, the DNA (STR) profiling was performed using the PowerPlex® 16 HS System from Promega (Sunnyvale, CA, USA) and data were crosschecked by DNA profiles available through http://www.dsmz.de.

### Isolation and cultivation of human bronchial epithelial cells

BE cells were isolated from proximal bronchi (main to segmental bronchi). After dissection and removal of adherent tissue, bronchi were rinsed with PBS and epithelial cells were gently scraped with a scalpel. Harvested cells were incubated in 25 cm^2^ culture flasks, additionally coated with collagen, in DMEM/F-12 growth medium (Gibco, Carlsbad, CA) containing 20% fetal calf serum (Biowest Ltd, Ringmer, UK), antibiotics (Pen/Strep) and antimycotic (Fungizone®, Gibco). One day later, cells were washed and grown in BEGM (bronchial epithelial cell growth medium) (Lonza, Basel, Switzerland) to sub-confluency. Cells at passages two to four were used for experiments. Homogeneity of cultured BE cells was analyzed by immunofluorescence staining for cytokeratin (≥98% of cells stained positive).

### Cell viability assay

Cell viability was assessed using the AlamarBlue® assay (Invitrogen, Life Technologies, Carlsbad, CA, USA), according to the manufacturer’s instructions. Cells were plated in triplicates in 96-well flat-bottom plates (10^4^ cells/well in 100 μl culture medium) and incubated 24 hours under normoxic or hypoxic conditions. Afterwards cells were incubated for 24, 48 or 72 hours with various concentrations of panobinostat, cisplatin or combination of both. Panobinostat was dissolved in DMSO (1 mM stock solution); thus, for the vehicle control DMSO was added to the cells at an amount corresponding to the DMSO amount in the highest panobinostat concentration used in the particular experiment. For fluorescence intensity measurements an excitation wavelength of 544 nm and an emission wavelength of 590 nm were used. Signals were read on a fluorescence microplate reader (FLUOstar Optima, BMG Labtech, Offenburg, Germany). The background fluorescence was determined by measuring the cell-free reactions and was afterwards subtracted from all values. Each experiment was performed at least three times with 3–5 measurements per sample and experiment.

### NSCLC tissue samples

Protein levels and mRNA were assessed in cryo-frozen NSCLC samples from 20 patients who were referred for surgical resection to the Division of Thoracic and Hyperbaric Surgery, Medical University of Graz. The study protocol was approved by the institutional ethics review board. Signed informed consent was obtained from all patients prior to surgery.

### Multicellular spheroids (MCS)

H23 cells were plated on ultra-low-adhesion, round-bottom, 96-well plates (7×10^3^ cells/well) and centrifuged at room temperature for 10 minutes at 1500 rpm. To enable MCS formation cells were incubated for 48 hours in humidified incubator under standard conditions (37°C, 5% CO_2_). The MCS were treated with single substances or combination of those for 24 h, followed by medium exchange. The cross-sectional areas of MCS were measured over 10 days on every second day and photos were made by using Olympus X51 Inverse system with a UPlanFI 4x objective, an Olympus XC50 camera system and Cell^F software (Olympus, Hamburg, Germany).

### RNA isolation and qRT-PCR

Total RNA was isolated from harvested cells or collected malignant and corresponding non-malignant tissue samples by using peqGOLD® Total RNA Kit (Peqlab, Erlangen, Germany) according to the manufacturer’s instructions. cDNA was reverse-transcribed from 0.5 μg of extracted RNA in a 20 μl reaction volume using the RevertAid^TM^ H Minus First Strand cDNA Synthesis Kit (Fermentas, St. Leon-Roth, Germany) with random hexamers as primers. RT conditions were as followed: 25°C for 10 minutes, 42°C for 60 minutes and 70°C for 10 minutes. For qRT-PCR 20 ng of each cDNA was used and reactions were performed by using following TagMan® assays on demand commercially available from Applied Biosystems: HDAC1, #Hs00606262_g1; HDAC2, #Hs00231032_m1; HDAC4, #Hs01041638_m1; HDAC5, #Hs00608366_m1; HDAC6, #Hs00195869_m1; HDAC8, #Hs00954353_g1; ß-actin, #Hs99999903_m1. All runs were performed on the Applied Biosystems 7900HT instrument. Amplification efficiency calculation was based on the slope of the standard curve (E = 10^-1/slope^-1) and was between 87% and 91% for all different HDACs. The ΔC_t_ values (C_t reference_ – C_t target_) for each target gene were calculated by using β-actin (ACTB) as a reference gene stably expressed in different samples.

### Hoechst staining

The nuclear fluorescent dye Hoechst 33342 (Invitrogen Molecular Probes, Oregon, USA) was used to visualize fragmented chromatin, an important morphological feature of apoptotic cells. H23 and A549 cells were seeded in 24-well plates at 2 × 10^5^ cells/well and treated with DMSO (vehicle control), panobinostat, cisplatin or with combination of both. Cells were cultured under normoxic or hypoxic conditions as already described. After 24 hours of treatment 0.5 μl/well Hoechst 33342 staining reagent was added and incubated for 5 minutes. Visualization was done by a digital imaging microscope Olympus Inverse X51. Three independent experiments were performed in triplicates for each condition. Per each well ten photographs were randomly made and the number of total cells (2000–3000 cells/condition) and apoptotic cells with fragmented chromatin was determined. Percentage of fragmented nuclei in relation to total number of nuclei was calculated.

### Fluorescent-activating cell sorting analyses (FACS)

In order to analyze cell cycle distribution and apoptosis-specific sub-G1 peak, propidium iodide (PI) staining combined with FACS analysis was used. Cells were seeded at a density of 10^5^/well in 12-well plates and 24 hours after seeding panobinostat and cisplatin were added either in combinations or as a single substance. Control cells were treated with DMSO at an amount corresponding to the highest panobinostat concentration used. 24 and 48 hours after drug-exposure floating cells in the culture medium were collected and adherent cells were harvested by trypsinization. Cells were pooled, collected by centrifugation at 1000 g for 5 minutes and washed once with PBS. Cells were resuspended in 50 μl PI lysis buffer (0.1% sodium citrate, 0.1% Triton X-100, 0.1 mg/ml RNAse A and 0.05 mg/ml PI). After 20 minutes incubation the DNA content was measured by flow cytometry (FACS Calibur, BD Biosciences, San Jose, USA). Results were analyzed with cell-cycle analysis software (ModFit LT 3.3 Software, Verity Software House, Topsham, ME). Caspase-3 Intracellular Activity Assay Kit I (PhiPhi-Lux® G1D2, Merck, Darmstadt, Germany) was used according to the manufacturer’s protocol. Shortly, approx. 3×10^5^ cells were incubated with 20 μl of the PhiPhiLux® substrate, incubated for 1 h at 37°C and washed once with PBS. The percentage of cells positive for active caspase-3 was determined by flow cytometry (FACS Calibur, BD Biosciences, San Jose, USA).

### Immunoblotting

Floating cells were collected and washed once with iced PBS. Adherent cells were harvested on ice by scraping in pre-cooled RIPA buffer and pooled with cells from supernatant. Samples were homogenized by sonication performed three times for 5 seconds on ice. Protein samples were separated by SDS-PAGE and transferred onto nitrocellulose membrane (BioRad Industries, Hercules, CA, USA). After staining with PonceauS and blocking with 5% non-fat milk or 5% BSA solution the membranes were incubated overnight at 4°C with specific primary antibodies. Incubations with secondary HRP-linked antibodies (anti-mouse 1:2000, anti-rabbit 1:2000) were performed for 1 hour at room temperature. For detection the membranes were incubated 5 minutes with enhanced chemiluminescence solution (SuperSignal West Pico Chemiluminescent Substrate; Thermo Scientific, Rockford, IL, USA). To reuse membranes for another antibody and for loading control, a stripping procedure with Restore Plus Western Blot stripping buffer (Thermo Scientific, Rockford, IL, USA) was performed. Blot pictures were cropped by using Photoshop CS5. Detailed ordering information and dilutions of specific antibodies are provided in Additional file 7.

### Duolink *in situ*fluorescence

H23 cells were grown on 8-well culture slides (3×10^4^ cells/well) for 72 hours in RPMI medium supplemented with 10% FCS. Cells were fixed in 4% formaldehyde for 20 minutes at 37°C, permeabilized with 0,1% Triton X-100 in PBS for 15 minutes at room temperature and blocked at room temperature for 30 min with 3% BSA in PBS and for additional 30 min at 37°C with blocking solution provided by producer. Primary antibodies (rabbit anti HDAC4 [#ABE262, Millipore]; mouse anti HIF-1α [#241809, R&D Systems]) were diluted 1:300 in antibody diluent provided by producer and incubated overnight at 4°C. PLA probes were diluted in provided antibody diluent and incubated for 1 h at 37°C, followed by 30 min ligation at 37°C, 100 min amplification at 37°C and detection (excitation = 594 nm; emission 624 nm). All steps were done in a pre-heated humidity chamber. Slides were counterstained with DAPI (excitation = 360 nm, emission = 460 nm), mounted, and analyzed on Olympus BX61VS microscope equipped with VS-ASW FL software for image analyses. The distance between two primary antibodies must be ≤30 nm to generate a signal in this assay.

### RNA silencing

H23 cells were seeded into 6-well plates (2×10^5^/well) and one day later transfected with specific siRNA (Thermo Scientific Dharmacon, Vienna, Austria) by using the jetPRIME^TM^ transfection reagent from Polypus-transfection (Illkirch, France). The ON-TARGETplus SMARTpools, containing four different siRNAs sequences, were used both for HIF-1α (# L-004018_00, 10 nM final concentration) and for HDAC4 (# L-003497_00, 20 nM final concentration). Transfection medium was replaced 24 hours later by normal growth medium and cells were incubated for additional 24 hours under normoxic or hypoxic conditions before analyzing the knock-down effects. Afterwards cells were treated with cisplatin (0, 16, 32 or 64 μM) for 24 hours and analyzed as required. Silencing effects were compared to cells transfected with corresponding concentration of non-silencing RNA (# D-001810-10-05) provided by the same producer. Specific gene-silencing effects were detected both by qRT-PCR and by immunoblotting. For qRT-PCR, assays on demand were used (HIF-1α, Hs00153153_m1; HDAC4, Hs01041638_m1; β-actin, Hs99999903_m1; Life Technologies Applied Biosystems, Vienna, Austria), reactions were prepared as described by producer and data were compared to β-actin (ACTB) as a housekeeping gene.

### Statistical analysis

GraphPad Prism version 5.0 (La Jolla, CA, USA) was used for graph drawing and statistical analyses. Data set comparisons were performed with Student’s t-test or Two-way ANOVA, as applicable. If not otherwise stated, all values are means ± SD. All tests were two-sided and *P* < 0.05 was considered to be statistically significant. Synergy in ability to reduce cell viability between cisplatin and panobinostat was calculated according to a Bliss independence model [52]. It was assumed that under additivity relative viabilities (cell viability in percent of vehicle control) of cisplatin alone (v_1_) and panobinostat alone (v_2_) are multiplied. Synergy for a drug combination is defined to be a relative viability (v_12_) that is lower than additivity, thus v_12_-v_1_*v_2_ < 0. The drugs act antagonistically, if relative viability is higher than additivity. Drug combination experiments were repeated six times, thus there were independent assessments of synergy that could be tested with the one-sample t-test. These calculations were performed separately for each dose combination.

## Electronic supplementary material

Additional file 1: **Expression of different class I and class II HDACs in three histological NSCLC subtypes.** Adenocarcinoma (n = 9), squamous cell carcinoma (n = 7), and large cell carcinoma (n = 4) samples were analyzed by qRT-PCR and different subtypes were compared. There seems to be a slight tendency of elevated expression levels in squamous cell carcinoma. However, statistical evaluation with Kruskal-Wallis test did not show any significant differences between NSCLC subtypes. (TIFF 35 KB)

Additional file 2: **Effects of co-treatment with cisplatin and panobinostat on cell viability.** Cells were treated with cisplatin or panobinostat alone and with combination of both substances. Cell viability was determined by AlamarBlue® assay. All results were compared to DMSO-treated cells set to 100%. NOX, normoxia; HOX, hypoxia; ns, not significant; * *P* < 0.05; ** *P* < 0.01; *** *P* < 0.001. (TIFF 25 KB)

Additional file 3: **Relative cell-viabilities and synergistic effects of cisplatin and panobinostat.** (DOC 46 KB)

Additional file 4: **Activation of apoptosis in A549 cells upon co-treatment with cisplatin and panobinostat.** Upon treatment A549 cells were stained with propidium iodide (PI) and analyzed by FACS for the sub-G1 peak indicating apoptosis. Data analysis was performed with the ModFit LT 3.3 software package and mean values are shown. NOX, normoxia; HOX, hypoxia; P, panobinostat; C, cisplatin. (TIFF 34 KB)

Additional file 5: **Cell cycle analysis of H23 and A549 cells.** Cells were treated with 16 nM panobinostat (Pano), 16 μM cisplatin (Cis) and with combination of both drugs. 24 hours latter cells were stained with propidium iodide for FACS analysis. The ModFit LT 3.3 software package was used for data analysis. Mean values of two independent experiments are shown. (TIFF 23 KB)

Additional file 6: **(A, B, C)** **Densitometric evaluation of immunoblotting data.** Experiments were performed as described for Figure 7 and relative intensities were determined by densitometric analysis of at least three blots. **(C)** HIF amount in DMSO-treated cells under HOX was set to 100%. **(D)** Immunoblotting for HIF in A549 cells. A549 cells treated with panobinostat (P, 16 nM), cisplatin (C, 16 μM) and combination of both (P + C), under normoxia (NOX) or hypoxia (HOX), were analyzed by immunoblotting. Relative intensities were determined by densitometric analysis and HIF amount in DMSO-treated cells under HOX was set to 100%. β-actin = loading control. (TIFF 32 KB)

Additional file 7: **List of antibodies used for immunoblotting.** (DOC 46 KB)
