# Supplementary figures and images for: Panobinostat reduces hypoxia-induced cisplatin resistance of non-small cell lung carcinoma cells via HIF-1α destabilization
Source: Mol Cancer. 2015 Jan 21;14:4. doi: 10.1186/1476-4598-14-4 (PMC4320451; doi:10.1186/1476-4598-14-4)

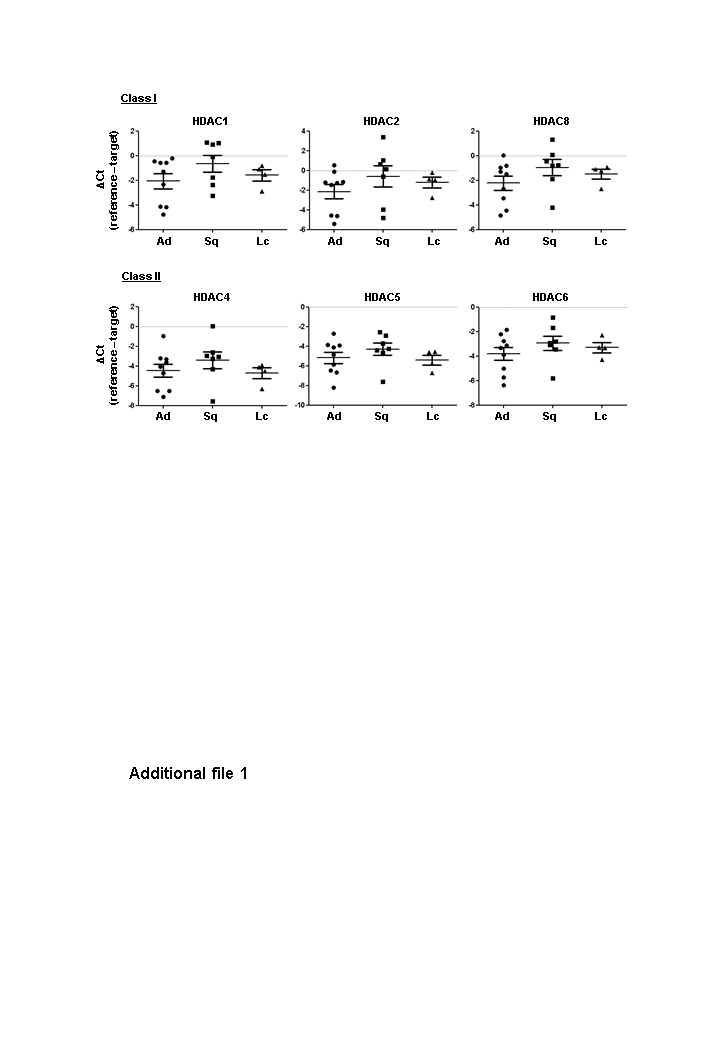

Supplement: Supplementary file 1 — Additional file 1: Expression of different class I and class II HDACs in three histological NSCLC subtypes. Adenocarcinoma (n = 9), squamous cell carcinoma (n = 7), and large cell carcinoma (n = 4) samples were analyzed by qRT-PCR and different subtypes were compared. There seems to be a slight tendency of elevated expression levels in squamous cell carcinoma. However, statistical evaluation with Kruskal-Wallis test did not show any significant differences between NSCLC subtypes. (TIFF 35 KB) [file 12943_2014_1468_MOESM1_ESM.tiff]

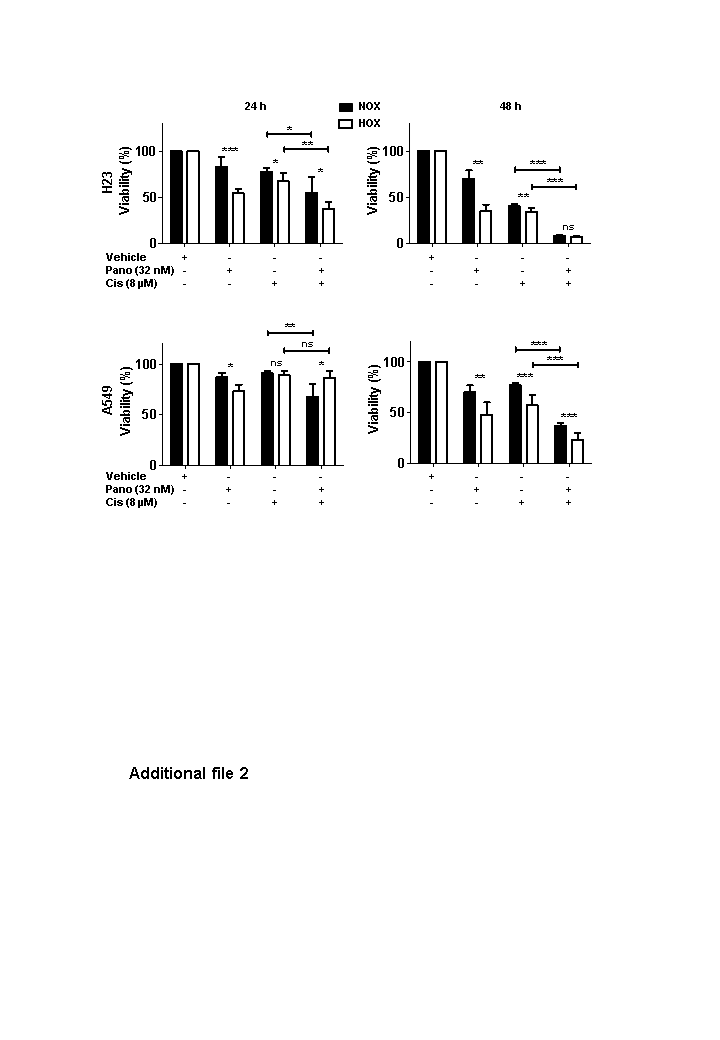

Supplement: Supplementary file 2 — Additional file 2: Effects of co-treatment with cisplatin and panobinostat on cell viability. Cells were treated with cisplatin or panobinostat alone and with combination of both substances. Cell viability was determined by AlamarBlue® assay. All results were compared to DMSO-treated cells set to 100%. NOX, normoxia; HOX, hypoxia; ns, not significant; * P < 0.05; ** P < 0.01; *** P < 0.001. (TIFF 25 KB) [file 12943_2014_1468_MOESM2_ESM.tiff]

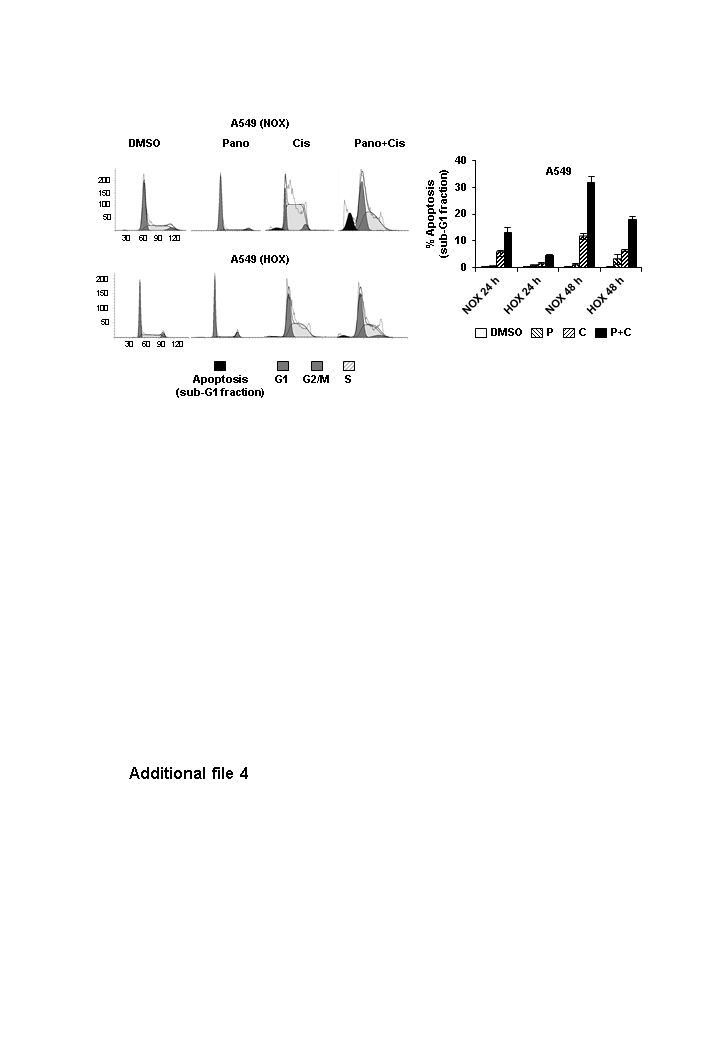

Supplement: Supplementary file 4 — Additional file 4: Activation of apoptosis in A549 cells upon co-treatment with cisplatin and panobinostat. Upon treatment A549 cells were stained with propidium iodide (PI) and analyzed by FACS for the sub-G1 peak indicating apoptosis. Data analysis was performed with the ModFit LT 3.3 software package and mean values are shown. NOX, normoxia; HOX, hypoxia; P, panobinostat; C, cisplatin. (TIFF 34 KB) [file 12943_2014_1468_MOESM4_ESM.tiff]

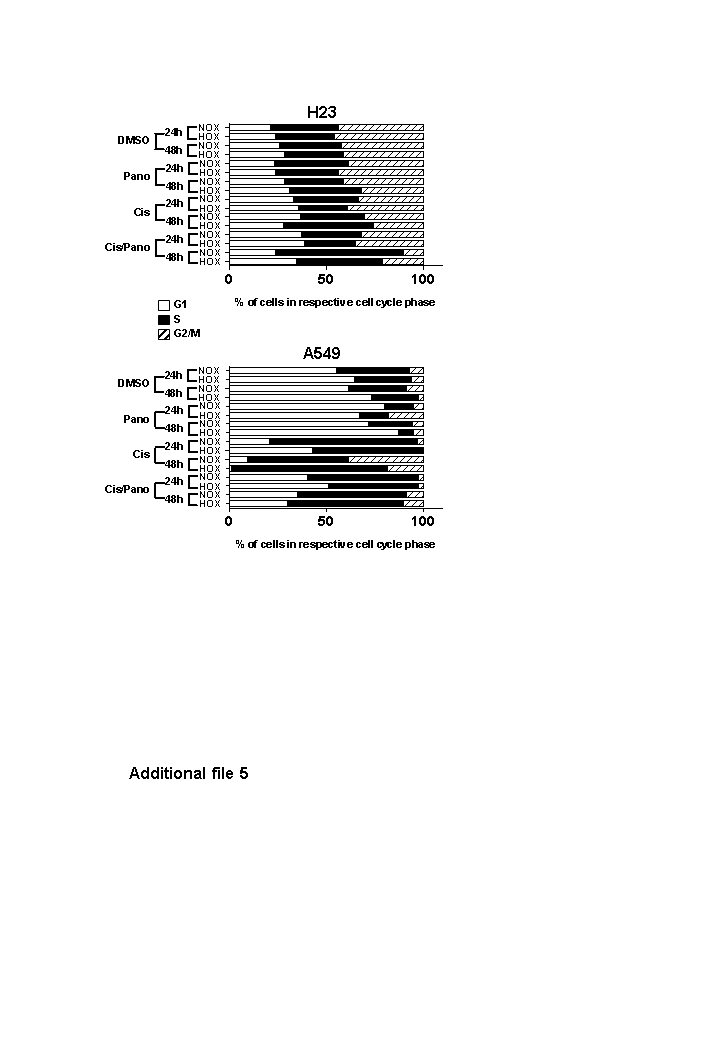

Supplement: Supplementary file 5 — Additional file 5: Cell cycle analysis of H23 and A549 cells. Cells were treated with 16 nM panobinostat (Pano), 16 μM cisplatin (Cis) and with combination of both drugs. 24 hours latter cells were stained with propidium iodide for FACS analysis. The ModFit LT 3.3 software package was used for data analysis. Mean values of two independent experiments are shown. (TIFF 23 KB) [file 12943_2014_1468_MOESM5_ESM.tiff]

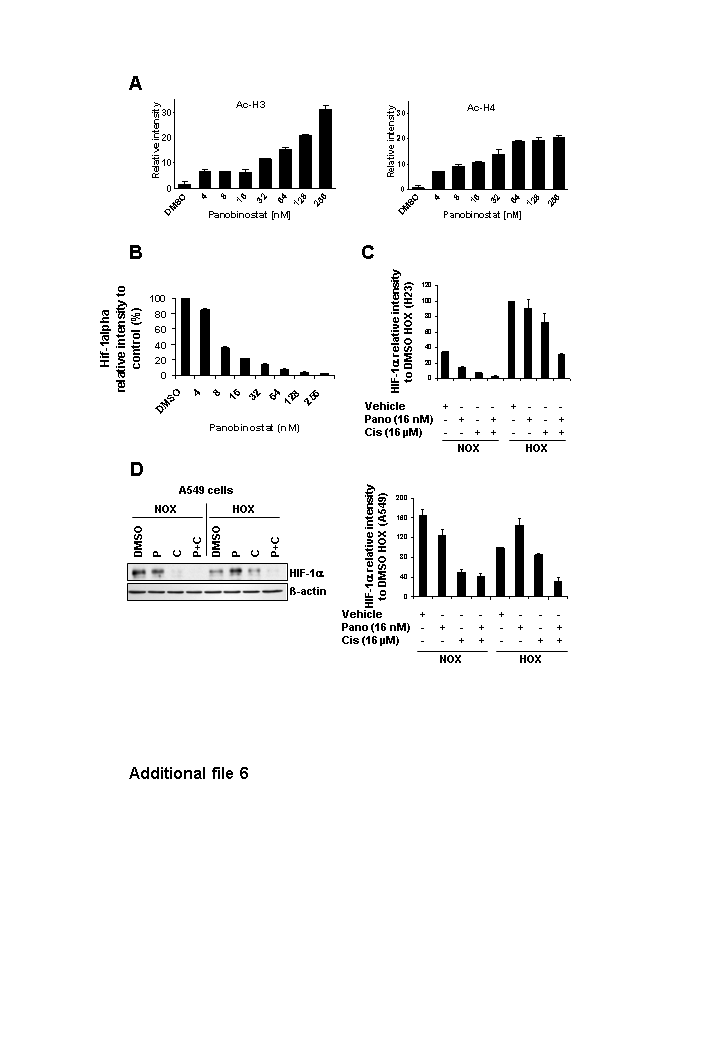

Supplement: Supplementary file 6 — Additional file 6: (A, B, C) Densitometric evaluation of immunoblotting data. Experiments were performed as described for Figure 7 and relative intensities were determined by densitometric analysis of at least three blots. (C) HIF amount in DMSO-treated cells under HOX was set to 100%. (D) Immunoblotting for HIF in A549 cells. A549 cells treated with panobinostat (P, 16 nM), cisplatin (C, 16 μM) and combination of both (P + C), under normoxia (NOX) or hypoxia (HOX), were analyzed by immunoblotting. Relative intensities were determined by densitometric analysis and HIF amount in DMSO-treated cells under HOX was set to 100%. β-actin = loading control. (TIFF 32 KB) [file 12943_2014_1468_MOESM6_ESM.tiff]
