# Supplementary material for: Panobinostat reduces hypoxia-induced cisplatin resistance of non-small cell lung carcinoma cells via HIF-1α destabilization
Source: Mol Cancer. 2015 Jan 21;14:4. doi: 10.1186/1476-4598-14-4 (PMC4320451; doi:10.1186/1476-4598-14-4)
Supplement: Supplementary file 3 — Additional file 3: Relative cell-viabilities and synergistic effects of cisplatin and panobinostat. (DOC 46 KB) [file 12943_2014_1468_MOESM3_ESM.doc]

**Additional file 3.** Relative cell-viabilities (drug/vehicle) and synergy effects of cisplatin (Cis) and panobinostat (Pano). Synergy of a drug combination (Cis + Pano) means that the relative cell viability of the drug combination is smaller than the product of the relative viabilities of single drugs, i.e. the difference between relative viability and the additive effect is negative (see statistical analysis).

| **Dose combination** |  |  | **Cis** | **Pano** | **Additive effect** | **Cis + Pano** | **Synergy** | **P-value** |
| --- | --- | --- | --- | --- | --- | --- | --- | --- |
| Cis 16 µM, Pano 16 nM | NOX | 24h | 0.42 ± 0.012 | 1.02 ± 0.032 | 0.41 ± 0.017 | 0.25 ± 0.006 | -0.16 ± 0.018 | 0.00028 |
|  |  | 48h | 0.11 ± 0.007 | 1.01 ± 0.019 | 0.11 ± 0.007 | 0.03 ± 0.001 | -0.08 ± 0.006 | 0.000045 |
|  | HOX | 24h | 0.52 ± 0.014 | 0.83 ± 0.036 | 0.43 ± 0.025 | 0.33 ± 0.030 | -0.10 ± 0.042 | 0.060 |
|  |  | 48h | 0.18 ± 0.007 | 0.80 ± 0.032 | 0.15 ± 0.011 | 0.04 ± 0.002 | -0.10 ± 0.012 | 0.00035 |
| Cis 8 µM, Pano 32 nM | NOX | 24h | 0.77 ± 0.019 | 0.82 ± 0.044 | 0.63 ± 0.035 | 0.54 ± 0.066 | -0.09 ± 0.068 | 0.25 |
|  |  | 48h | 0.39 ± 0.014 | 0.71 ± 0.039 | 0.28 ± 0.013 | 0.08 ± 0.004 | -0.20 ± 0.014 | 0.000035 |
|  | HOX | 24h | 0.67 ± 0.034 | 0.53 ± 0.009 | 0.36 ± 0.021 | 0.36 ± 0.017 | 0.00 ± 0.035 | 0.996 |
|  |  | 48h | 0.33 ± 0.017 | 0.36 ± 0.030 | 0.12 ± 0.015 | 0.07 ± 0.004 | -0.05 ± 0.014 | 0.013 |

NOX, normoxia; HOX, hypoxia
