# Supplementary material for: Panobinostat reduces hypoxia-induced cisplatin resistance of non-small cell lung carcinoma cells via HIF-1α destabilization
Source: Mol Cancer. 2015 Jan 21;14:4. doi: 10.1186/1476-4598-14-4 (PMC4320451; doi:10.1186/1476-4598-14-4)
Supplement: Supplementary file 7 — Additional file 7: List of antibodies used for immunoblotting. (DOC 46 KB) [file 12943_2014_1468_MOESM7_ESM.doc]

**Additional file 7.** List of antibodies used for immunoblotting.

| **1^st^**  **antibody** | **Gel**  **(%)** | **Catalogue**  **Nr.** | **Company** | **Dilution** | **Species** |
| --- | --- | --- | --- | --- | --- |
| Acetylated H3 | 15 | 8655 | Santa Cruz | 1:1000 | rabbit |
| Acetylated H4 | 15 | 8660 | Santa Cruz | 1:1000 | rabbit |
| Cl. Caspase-3 | 15 | 9664 | Cell Signaling | 1:1000 | rabbit |
| Cl. Caspase-7 | 15 | 9491 | Cell Signaling | 1:1000 | rabbit |
| PARP | 10 | 9542 | Cell Signaling | 1:1000 | mouse |
| HDAC1 | 10 | MAB1536 | R&D Systems | 1:500 | mouse |
| HDAC4 | 10 | 2072 | Cell Signaling | 1:1000 | rabbit |
| HDAC5 | 6 | 133225 | Santa Cruz | 1:1000 | mouse |
| HDAC6 | 6 | SP7084P | Acris Antibodies | 1:500 | rabbit |
| HDAC7 | 6 | AP00276PU-N | Acris Antibodies | 1500 | rabbit |
| HDAC9 | 6 | 398003 | Santa Cruz | 1:500 | mouse |
| HIF-1α | 10 | 610958 | BD Biosciences | 1:500 | mouse |
| HIF-2α | 10 | 46691 | Santa Cruz | 1:2000 | mouse |
| ß-actin | Var. | 47778 | Santa Cruz | 1:5000 | mouse |

Cl. = cleaved; Var. = variable (depending on original SDS-PA gel)
